# 193. Trends in mortality caused by Enterocolitis due to Clostridium difficile among United States population: A CDC WONDER Database Study from 1999-2023

**DOI:** 10.1093/ofid/ofaf695.068

**Published:** 2026-01-11

**Authors:** Muhammad Sohaib Asghar, Rupesh Andani, Maria Duharte, Afsana Ansari Shaik, Shehar Bano, Luis Duharte-Vidaurre

**Affiliations:** AdventHealth Sebring, Sebring, FL; AdventHealth Sebring, Sebring, FL; South Florida State College, Tampa, Florida; Mayo Clinic, Rochester, Minnesota; AdventHealth Sebring, Sebring, FL; AdventHealth Sebring, Sebring, FL

## Abstract

**Background:**

*Clostridium difficile* infection (CDI) remains a significant public health concern in the United States, contributing to considerable morbidity and mortality, particularly among hospitalized and elderly populations. This study examines the temporal trends in CDI-related mortality across the United States over recent decades.

**Figure 1A:**
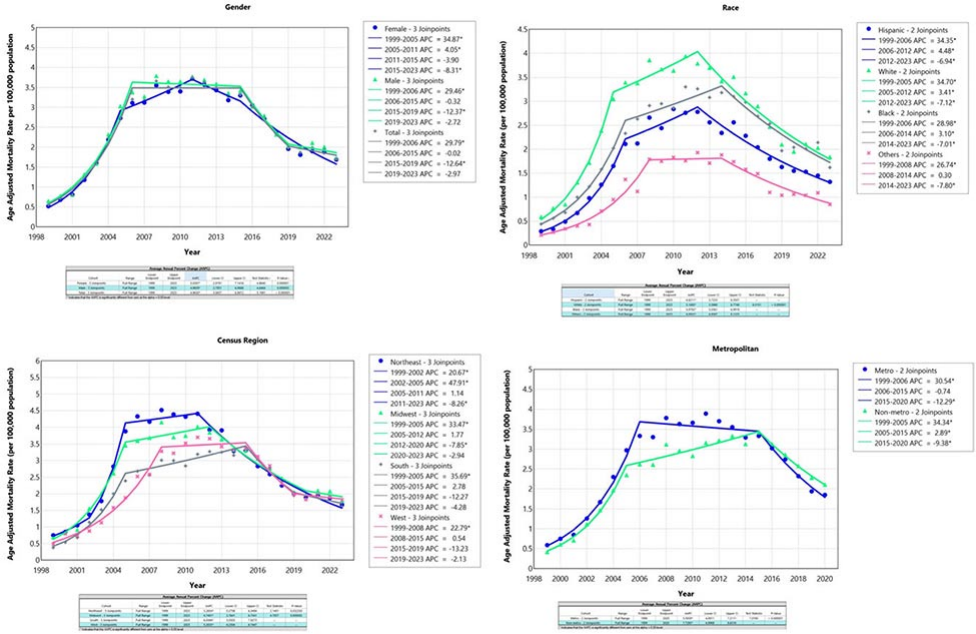
Mortality trends among gender; B: Race; C: US Census Region; D: Metropolitan areas.

Mortality ratio (Gender):

Female = 58.15%

Male = 41.85%

Mortality ratio (Race):

Hispanic = 5.53%

Whites = 83.92%

Blacks = 8.12%

Others = 2.22%

Mortality ratio (US Census Region):

Northeast = 22.18%

Midwest = 24.36%

South = 33.13%

West = 20.33%

Mortality ratio (Metropolitan):

Metropolitan areas: 83.78%

Non-metropolitan areas: 16.22%

**Figure 2A:**
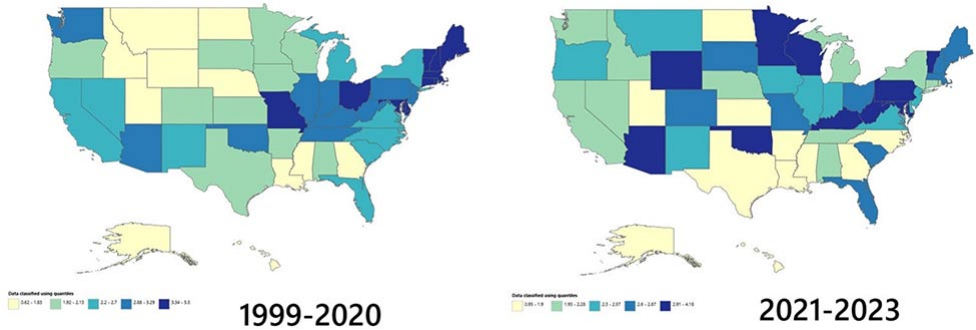
Trends in mortality rates caused by Clostridium Difficile in United States from 1999-2020; and B: from 2021-2023.

Top 90th percentile States (crude mortality rate per 100,000 population):

Rhode Island: 6.55

Maine: 4.61

Ohio: 4.58

Vermont: 4.21

West Virginia: 4.18

Missouri: 4.11

Bottom 10th percentile States (crude mortality rate per 100,000 population):

Georgia: 1.59

Mississippi: 1.49

Louisiana: 1.32

Utah: 1.26

Alaska: 0.96

Hawaii: 0.77

**Methods:**

In CDC WONDER analysis, we used total US residents of any age from all 50 states who died due to CDI spanning from the years 1999-2023. The dataset employed demographic attributes, place of death, state-level information, as well as classification into census regions, and the distinction between metropolitan and non-metropolitan localities. The ethnic/racial groups used in our study were classified as Hispanic, Black/African American, White, and others (including American Indian/Alaskan Native, Asian/Pacific Islander) individuals as indicated on the death certificates. US Census regions were categorized into Northeast, Midwest, South, and West based on the definitions provided by the Census Bureau.

**Figure 3:**
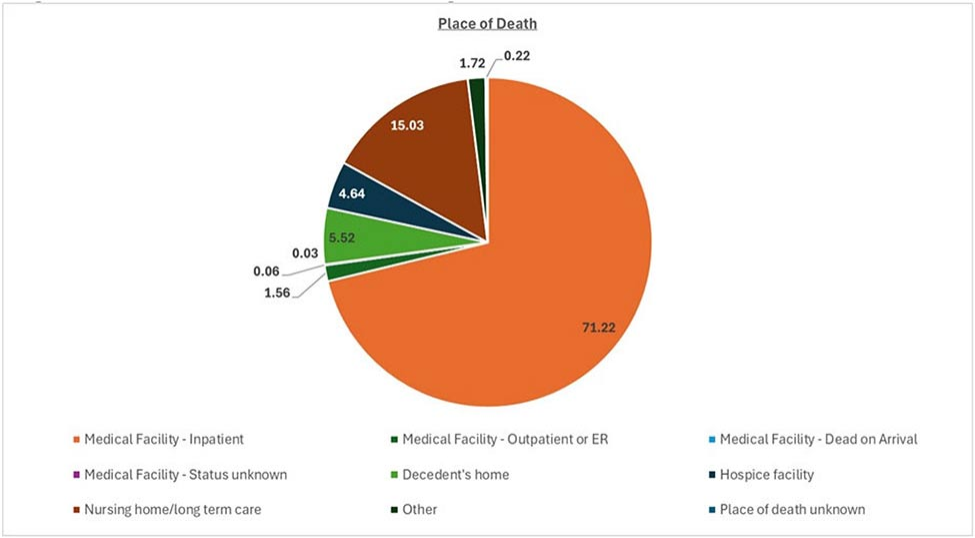
Place of death among individuals infection with clostridium difficile.

Place of Death:

Medical Facility - Inpatient: 71.22%

Medical Facility - Outpatient or ER: 1.56%

Medical Facility - Dead on Arrival: 0.06%

Medical Facility - Status unknown: 0.03%

Decedent's home: 5.52%

Hospice facility: 4.64%

Nursing home/long term care: 15.03%

Other: 1.72%

Place of death unknown: 0.22%

**Figure 4:**
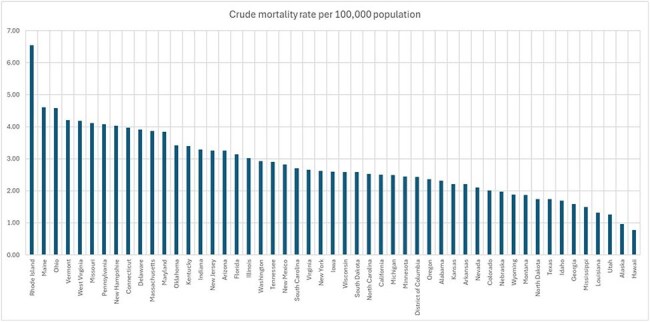
Crude mortality rate among States.

Crude mortality rate is represented per 100,000 population from the years 1999 to 2023.

**Results:**

There were a total of 216,311 deaths associated with CDI over the study span. Out of them, 58.1% were females, 41.9% were males, 83.9% were White individuals, 8.12% were Black/African Americans, 5.53% were Hispanics, and 2.22% belonged to other racial groups. About 33% (one-thirds) of the deaths occured in South region followed by 24.4% in Midwest, 22.2% in Northeast and 20.3% in West region. According to NCHS rural-urban scheme classification, 83.8% deaths occured in metropolitan areas and the rest 16.2% in non-metropolitan areas.

**Conclusion:**

National surveillance data indicate a marked increase in CDI-associated deaths during the early 2000s, largely driven by the emergence of hypervirulent strains and increased antibiotic resistance. However, recent years have shown a gradual decline in mortality rates, attributed to improved infection control practices, antimicrobial stewardship programs, and heightened public awareness. Despite these gains, disparities persist among racial, geographic, and age-specific subgroups, highlighting the need for targeted interventions. This underscores the evolving landscape of CDI mortality and the importance of sustained preventive strategies to further reduce the burden of this infection.

**Disclosures:**

All Authors: No reported disclosures

